# Enhanced Hippocampal Long-Term Potentiation and Fear Memory in *Btbd9* Mutant Mice

**DOI:** 10.1371/journal.pone.0035518

**Published:** 2012-04-19

**Authors:** Mark P. DeAndrade, Li Zhang, Atbin Doroodchi, Fumiaki Yokoi, Chad C. Cheetham, Huan-Xin Chen, Steven N. Roper, J. David Sweatt, Yuqing Li

**Affiliations:** 1 Interdisciplinary Program in Biomedical Sciences, College of Medicine, University of Florida, Gainesville, Florida, United States of America; 2 Department of Neurology, College of Medicine, University of Florida, Gainesville, Florida, United States of America; 3 Center for Neurodegeneration and Experimental Therapeutics, Department of Neurology, School of Medicine, University of Alabama at Birmingham, Birmingham, Alabama, United States of America; 4 Department of Neurosurgery, College of Medicine, University of Florida, Gainesville, Florida, United States of America; 5 Department of Neurobiology, School of Medicine, University of Alabama at Birmingham, Birmingham, Alabama, United States of America; University of Turin, Italy

## Abstract

Polymorphisms in *BTBD9* have recently been associated with higher risk of restless legs syndrome (RLS), a neurological disorder characterized by uncomfortable sensations in the legs at rest that are relieved by movement. The BTBD9 protein contains a BTB/POZ domain and a BACK domain, but its function is unknown. To elucidate its function and potential role in the pathophysiology of RLS, we generated a line of mutant *Btbd9* mice derived from a commercial gene-trap embryonic stem cell clone. *Btbd9* is the mouse homolog of the human *BTBD9*. Proteins that contain a BTB/POZ domain have been reported to be associated with synaptic transmission and plasticity. We found that Btbd9 is naturally expressed in the hippocampus of our mutant mice, a region critical for learning and memory. As electrophysiological characteristics of CA3-CA1 synapses of the hippocampus are well characterized, we performed electrophysiological recordings in this region. The mutant mice showed normal input-output relationship, a significant impairment in pre-synaptic activity, and an enhanced long-term potentiation. We further performed an analysis of fear memory and found the mutant mice had an enhanced cued and contextual fear memory. To elucidate a possible molecular basis for these enhancements, we analyzed proteins that have been associated with synaptic plasticity. We found an elevated level of dynamin 1, an enzyme associated with endocytosis, in the mutant mice. These results suggest the first identified function of Btbd9 as being involved in regulating synaptic plasticity and memory. Recent studies have suggested that enhanced synaptic plasticity, analogous to what we have observed, in other regions of the brain could enhance sensory perception similar to what is seen in RLS patients. Further analyses of the mutant mice will help shine light on the function of BTBD9 and its role in RLS.

## Introduction

Recently, two genome-wide association studies (GWAS) have correlated polymorphisms in the *BTBD9* gene with restless legs syndrome (RLS) [1], [2], a neurological disorder characterized by unpleasant sensations in the legs at rest that are relieved by movement [3]. The *BTBD9* gene encodes a protein with two structurally conserved domains. One domain is a bric-á-brac, tramtrack, broad complex (BTB)/pox virus and zinc finger (POZ) domain near the N-terminus, which has been associated in other proteins with transcriptional regulation, cytoskeleton dynamics, ion conductance, and protein ubiquitination [4]. The other domain is a BTB and C-terminal kelch (BACK), which has been associated in other proteins with maintaining proper substrate orientation, primarily for E3 ubiquitin-ligase complexes [5].

Previous studies have shown that the mRNA of the *Btbd9* gene, the mouse homolog of the human *BTBD9* gene, was down-regulated in a model of Lesch-Nyhan syndrome [6], a neurological disorder sometimes referred to as juvenile gout; and in an embryonic stem cell line that lacks the amyloid precursor proteins App and Aplp2, which are involved in synaptic formation and repair [7], [8]. Contrastingly, in baboons that consumed moderate and high levels of docosahexaenoic acid, which is an important fatty acid in the central nervous system and during development, *BTBD9* mRNA was up-regulated [9]–[11]. Additionally, *Btbd9* gene expression has been correlated with the level of iron in the midbrain of an inbred strain of mice [12]. Finally, previous studies using *in situ* hybridization showed that *Btbd9* mRNA was expressed in several brain regions, including the thalamus, hypothalamus, cortex, striatum and hippocampus, and also expressed in all levels of the spinal cord [13]. All of these studies, however, have been limited in scope in examining the function of the *BTBD9* protein *in vivo* and its potential role in the pathophysiology of RLS.

Proteins containing similar domains to BTBD9 have been implicated in a variety of aspects of synaptic plasticity and transmission. For instance, abrupt, a BTB-zinc finger protein in *Drosophila melanogaster*, has been shown to be involved in proper motor neuron targeting and dendritic branching [14], [15]. Additionally, overexpression of kelch, a BTB-Kelch protein in *D. melanogaster*, led to an increase in dendritic branching, while a loss of kelch inhibited dendritic branching [16]. KEL-8, another BTB-Kelch protein in the nematode *Caenorhabditis elegans*, was shown to be a critical component of ubiquitin-mediated turnover of the glutamate receptor GLR-1 [17]. Similarly, actinfilin, a brain-specific BTB-Kelch protein in rats, was shown to regulate the ubiquitin-mediated turnover of the glutamate receptor GLR-6 [18], [19].

We attempted to elucidate the function of Btbd9 and its potential role in the pathophysiology of RLS by creating a knockout of the *Btbd9* gene in mice. We found that the Btbd9 protein was naturally expressed in the hippocampus, a brain region critical for learning and memory. Additionally, a study of non-medicated RLS patients showed minor increases in gray matter density of the ventral hippocampus [20], but the consequential effects were not investigated. As the CA3-CA1 Schaffer collateral synapses of the hippocampus are well characterized using electrophysiological techniques and are glutamate-mediated, we analyzed the homozygous *Btbd9* mutant mice in this region. We found that the *Btbd9* mutant mice exhibited enhanced long-term potentiation (LTP) and paired-pulse ratios (PPRs). Furthermore, we have found that the *Btbd9* mutant mice had an enhanced cued and contextual fear memory. Lastly, we found that the *Btbd9* mutant mice had an increase in levels of dynamin 1, an enzyme associated with endocytosis. Therefore, our data, taken together, suggest that the first known function of the Btbd9 protein is that it is involved in regulating hippocampal synaptic plasticity and learning and memory.

## Materials and Methods

All experiments were carried out in compliance with the USPHS Guide for Care and Use of Laboratory Animals and approved by the Institutional Animal Care and Use Committee at the University of Alabama at Birmingham (Protocol # 090908921).

### Generation of the *Btbd9* knockout mice

**Figure 1:**
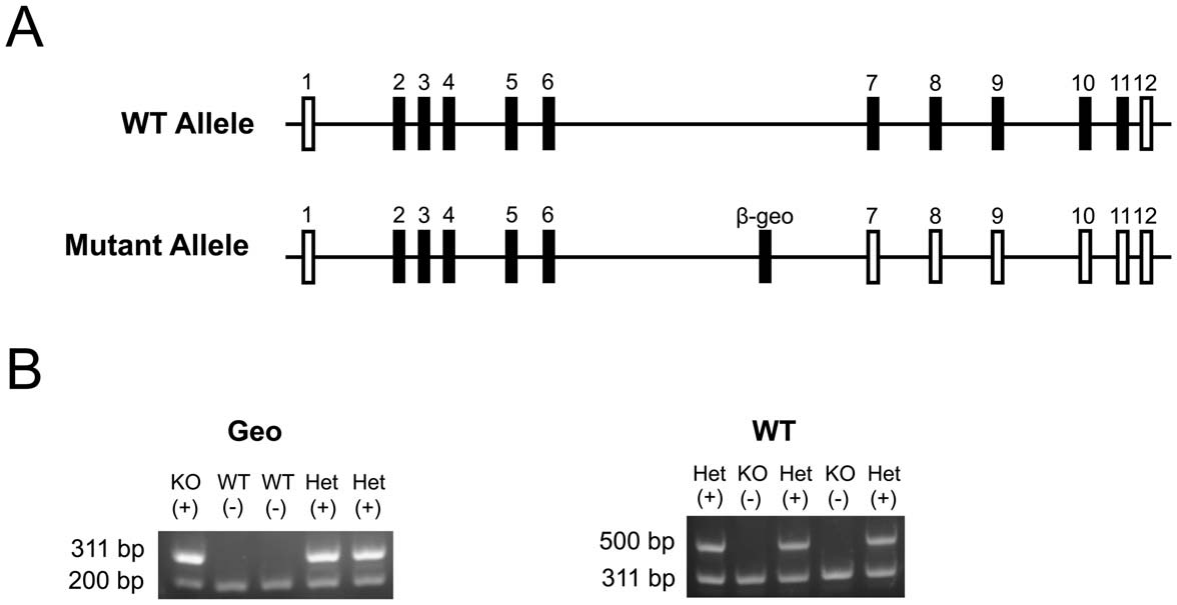
Generation and genotyping of *Btbd9* mutant mice. (**A**) The normal wild-type (WT) allele of the *Btbd9* gene contains 12 exons. A commercially available mutant allele of the *Btbd9* gene has a β-geo gene trap inserted into the sixth intron. Filled vertical rectangles represent coding exons; open vertical rectangles represent non-coding exons. (**B**) To genotype the mice a two-step process was taken. First, primers were used to detect specifically the β-geo gene trap vector, which produced a fragment of approximately 311 bp in length with an internal control fragment of approximately 200 bp in length. Second, primers were used to detect specifically a WT allele, which would produce a fragment of approximately 500 bp in length and an internal control β-geo fragment of 311 bp in length.

We obtained a commercial embryonic stem (ES) cell clone that harbored a β-geo gene trap vector in the sixth intron of the *Btbd9* gene (RRE078, BayGenomics). The gene trap was approximately 8.5 kilobase pairs (kb) long and included a single exon of the *Engrailed-2* (*En2*) gene fused to coding regions of the bacterial *lacZ* and *neomycin phosphotransferase II* genes. The *En2* exon contained a 5′-splice site, while at the end of the β-geo gene trap there was a polyadenylation (pA) signal sequence. These two sequences, the splice acceptor site and the pA signal sequence, created a new exon within the sixth intron of the *Btbd9* gene, which should terminate transcription at the end of the gene trap, and produce a fusion protein that contains the N-terminal portion of the Btbd9 protein and a β-galactosidase/neomycin fusion protein. The ES cells were expanded and its fusion mRNA verified by RT-PCR (data not shown). After insertion of the expanded ES cells into blastocysts, they were implanted into pseudopregnant females, and chimeras were generated. Chimeras that successfully transmitted to germline the mutated *Btbd9* gene and passed it to their offspring were detected by PCR using primers designed to target and amplify a sequence within the gene trap vector (v1531 – 5′-GGTCCCAGGTCCCGAAAACCAAAGAAGA-3′ and v1842R – 5′-ACAGTATCGGCCTCAGGAAGATCGC-3′). Heterozygous mutant mice were interbred to generate homozygous mutant mice, which were identified initially using a microsatellite marker approximately 4 megabase pairs from the *Btbd9* gene, which can identify differences between mouse inbred strains (D17M100a – 5′-GTTAAGAATGATTTTCACACTACAAGA-3′ and D17M100b – 5′-AGCACATGTACTTACTCATATACGTGC-3′). The size of PCR products from 129/SvJ strain is 129 bp, which represents that of the mutant allele from the ES cell, while those from C57BL6J is 119 bp, which represents that of the wild-type allele from the backcross. To determine the location of the gene trap vector within the sixth intron of the *Btbd9* gene, we used long and accurate (LA)-PCR. With LA-PCR we were able to successfully amplify sequences up to approximately 20 kb, which was important as the length of the sixth intron of the *Btbd9* gene is approximately 179.2 kb long. We synthesized primer sets at approximately 10 kb intervals within the sixth intron (Table S1), and then serially amplified approximately 10 kb fragments (Figure S1A) and screened for failure of LA-PCR reactions using genomic template DNA isolated from the homozygous mutant mice. We were able to narrow down the approximate location of the gene trap in the sixth intron to a 13.2 kb region between approximately 89.3 kb and 102.5 kb from the start of the sixth intron. Next, we synthesized primer sets at approximately 500 bp intervals in this region and conducted PCR (Table S1). This narrowed the possible insertion site further to 102,000 to 102,501 bp from the start of the sixth intron (Figure S1B). Lastly, we generated a primer set located at 102,200 bp from the start of the sixth intron and conducted PCR (Table S1). This narrowed the insertion site to between 102,200 and 102,501 bp from the start of the sixth intron (Figure S1B). Mice were backcrossed to the C57BL/6 background. Mating was conducted by interbreeding heterozygous *Btbd9* mutant mice to generate experimental mice. Mice were genotyped by PCR using a primer set designed to detect the wild-type strain (102000A – 5′-ctgagatgattaacaagagctgagggct-3′ and 6ERA – 5′-tcagccacgtcttctaaatgtaatggtt-3′, Figure 1B, Figure S1C) and a primer set designed to detect the mutant strain (v1531 and v1842R, Figure 1B, Figure S1C, Table S2) together with a set of primers to serve as internal controls (102000B – 5′- AGATGATTAACAAGAGCTGAGGGCT -3′ and 102200R – 5′-ACACTAAGCTTTCCCACGGGTGCACAT-3′). In all experiments, adult homozygous *Btbd9* mutant mice and littermate wild-type (WT) control C57BL/6 mice were used.

### RT-PCR

mRNA was obtained from 2 WT and 2 homozygous *Btbd9* mutant mice brains using a Qiagen RNeasy Protect Kit. RT-PCR was performed using protocols from Qiagen OneStep RT-PCR kit with the forward primer located in the exon before the intron containing the gene-trap (Exon6 – 5′-TTGAAGTGTCCATGGACGAACTTGATTGGA-3′) and the reverse primer located in the exon after this intron (Exon7R – 5′-GAAAATCTTGTTCACTGTGTTGTGAGTCC-3′).

### Electrophysiology: field recordings

Recordings were conducted from 6 WT and 6 homozygous *Btbd9* mutant mice. Preparation of hippocampal slice and electrophysiological analysis were performed as described previously [21]–[23]. In brief, hippocampi of adult mutant or wild type mice were rapidly removed and briefly chilled in ice-cold cutting saline (110 mM sucrose, 60 mM NaCl, 3 mM KCl, 1.25 mM NaH_2_PO_4_, 28 mM NaHCO_3_, 5 mM D-glucose, 500 µM CaCl_2_, 7 mM MgCl_2_, and 600 µM ascorbate). Transverse slices 400-µm thick were prepared with a Vibratome and maintained at least 45 min in a holding chamber containing 50% artificial cerebral spinal fluid (aCSF) (125 mM NaCl, 2.5 mM KCl, 1.25 mM NaH_2_PO_4_, 25 mM NaHCO_3_, 25 mM D-glucose, 2 mM CaCl_2_, and 1 mM MgCl_2_) and 50% cutting saline. The slices were then transferred to a recording chamber and perfused (1 mL/min) with 100% aCSF. Slices were allowed to equilibrate for 60–90 min in a Fine Science Tools interface chamber at 30°C. All solutions were continuously bubbled with 95% O_2_/5% CO_2_. For extracellular field recordings, glass recording electrodes were filled with aCSF and placed in the stratum radiatum of the CA1 hippocampal region. Test stimuli were delivered to the Schaffer collateral/commissural pathway with bipolar Teflon coated platinum stimulating electrode positioned in the stratum radiatum of the CA3 hippocampal region. Responses were recorded using a computer with AxoClamp pClamp 8 data acquisition software. Excitatory post-synaptic potential (EPSP) slope measurements were taken after the fiber volley to eliminate contamination by population spikes. Following at least 20 min of stable baseline recording, long-term potentiation (LTP) was induced with two, 100 Hz tetani (1 second), with an interval of 20 seconds between tetani. Synaptic efficacy was monitored by recording fEPSPs every 20 seconds beginning 0.5 hr prior to induction of LTP and ending 3 hr after induction of LTP (traces were averaged for every 2 min interval). Paired pulse ratios (PPRs) were measured at various inter-stimulus intervals (10, 20, 50, 100, 150, 200, 250, and 300 ms). All experimental stimuli were set to an intensity that evoked 50% of the maximum field EPSP (fEPSP) slope. To measure input-output relationships, test stimuli were delivered and responses recorded at 0.05 Hz with every six consecutive responses over a 2 min period pooled and averaged. fEPSPs were recorded in response to increasing intensities of stimulation (from 2.5 µA to 45.0 µA).

### Electrophysiology: whole-cell recordings

Recordings were conducted from 3 juvenile WT mice and 2 homozygous *Btbd9* mutant littermates. Animals were anesthetized by the inhalation of isoflurane, decapitated and the brain was rapidly removed. 400 µm-thick coronal brain slices were cut with a Vibratome (Technical Products International, St. Louis, MO). Slices were incubated on cell culture inserts (8 µm pore diameter, Becton Dickinson, Franklin Lakes, NJ) covered by a thin layer of artificial cerebrospinal fluid (ACSF) containing (in mM) 124 NaCl, 26 NaHCO_2_, 1.25 NaH_2_PO_4_, 2.5 KCl, 1 CaCl_2_, 6 MgCl_2_, 10 D-glucose and surrounded by a humidified 95% O_2_ and 5% CO_2_ atmosphere at room temperature (22°C). After at least 1 hr incubation, the slice was transferred to a submerged recording chamber with continuous flow (2 ml/min) of ACSF as described above except for 2.4 mM CaCl_2_ and 1.3 mM MgCl_2_ and gassed with 95% O_2_-5% CO_2_ giving pH 7.4. All experiments were carried out at 30°C. Whole-cell recordings were made from pyramidal cells in the hippocampal CA1 region using infrared-differential interference contrast microscopy and an Axopatch 1D amplifier (Axon Instruments, Foster City, CA). Patch electrodes had a resistance of 3–5 MΩ when filled with intracellular solution containing (in mM): 125 K-gluconate, 8 NaCl, 10 HEPES, 4 MgATP, 0.3 Na3GTP, 0.2 EGTA, and 0.1% biocytin (pH 7.3 with KOH, osmolarity 290–300 mOsM). mEPSCs were recorded at −68 mV and with the both solution containing 1 µM TTX(a sodium channel blocker, 50 µM AP5(an NMDA receptor blocker) and 50 µM picrotoxin (a blocker of GABA receptor). Series resistance was 9–15 MΩ and cells were rejected if it changed >10% throughout the recording session. All drugs were purchased from Sigma-Aldrich. Data were acquired using pClamp 10 software. The recordings were started 5–10 min after accessing the cell to allow for stabilization of spontaneous synaptic activity. Analysis of mEPSCs was based on 5 min continuous recordings from each cell. Events were detected using the Mini Analysis Program (Synaptosoft) with parameters optimized for each cell and then visually confirmed prior to analysis. The peak amplitude, 10–90% rise time and the decay time constant were measured based on the average of all events aligned by rise phase.

### Fear conditioning

Memory formation and recall was assessed by contextual and cued fear conditioning test as previously described [22], [24]. In brief, 20 WT mice and 15 homozygous *Btbd9* mutant mice were trained during the morning of the first day in contextual chambers with wire shock grid floors. Mice were first allowed to explore the context for four min before presentation of the first 30 seconds tone at 90 dB, which co-terminated with a 1 second, 0.5 mA electrical foot shock. Two tone-shock pairings were presented in total with a shock interval of 120 seconds. Mice remained in the chamber for an additional 60 seconds before being returned to their home cages. The chamber was cleaned with 70% ethanol between animals. The next morning mice were monitored in the context chambers for their freezing behavior for 5 min, without the presentation of tone or shock. In the afternoon, the attributes of the environment were significantly altered. Mice were then allowed to explore this novel environment for 3 min before presentation of the tone, without shock, for 3 min. The chamber was cleaned with 75% isopropanol between animals. Response to the electric shocks during the training phase was conducted by an investigator blind to the genotypes of the mice, on two rating scales. The first scale measured the gross response to the electric shock, and a score of 1 to 3 was given, where 1 represented no response, 2 represented flinching or modest interrupting in ongoing behavior but did not generate any gross movement, and 3 represented running or jumping or major interruption in ongoing behavior that generated gross movements. As all mice in the fear conditioning experiment responded with level 3 responses, the investigator then rated the response on a scale of 1 to 5, where 1 is a minimal but gross movement in response to the shock and 5 is a very large gross movement in response to the shock. The time each mouse spent without any movement during the test was counted as freezing behavior. Freezing behavior was monitored and recorded by video and evaluated by software (Video Freeze, Med Associates). The freezing time (seconds) was divided by session time (for context, 5 min; for cued, 3 min) and expressed as percent freezing. Finally, we measured electrical shock pain threshold as previously described [25]. In brief, we systematically applied increased shock intensities from 0.1 mA to 1.0 mA to 7 WT mice and 6 homozygous *Btbd9* mutant mice. Intensities at which the mouse flinched, jumped, or vocalized were recorded and analyzed.

### Western blot

Two types of fractions were used depending on the experiment. The first was a whole hippocampus fraction and the second was a synaptosomal fraction, both from 3 WT mice and 3 homozygous *Btbd9* mutant mice. In both cases, the hippocampi were dissected and quickly frozen in liquid nitrogen. Whole hippocampus fraction preparation was performed as previously described [23], [26], [27]. In brief, the hippocampi were then homogenized in 400 µL of ice-cold lysis buffer [50 mM Tris·Cl (pH 7.4), 175 mM NaCl, 5 mM EDTA, Complete Mini Protease Inhibitor Cocktail (Roche)] and sonicated for 10 seconds. One-ninth volume of 10% Triton X-100 in lysis buffer was added to the homogenates. The homogenates were incubated for 30 min on ice, and then centrifuged at 10,000× g for 15 min at 4°C. The supernatants were then collected and the protein concentration was measured by Bradford assay with bovine serum albumin as standards. The homogenates were mixed with SDS-PAGE loading buffer and boiled for 5 min, incubated on ice for 1 minute, and then centrifuged for 5 min to obtain the supernatant for loading at 40 µg each lane. Synaptosomal fractions were prepared as previously described [26], [28]. In brief, the hippocampi were defrosted in 5 mL of ice-cold TEVP buffer [10 mM Tris-Cl (pH 7.4), 5 mM NaF, 1 mM Na_3_VO_4_, 1 mM EDTA, 1 mM EGTA] containing 320 mM sucrose for 5 min and homogenized. The homogenates were centrifuged for 10 min at 800× g at 4°C and the supernatants were collected. Supernatants were then centrifuged for 15 min at 9,200× g and the pellets were obtained. After briefly rinsing with 1 mL TEVP buffer containing 35.6 mM sucrose, pellets were resuspended in 2 mL TEVP buffer containing 35.6 mM sucrose and put on ice for 30 min. The samples were vortexed and centrifuged for 20 min at 25,000× g at 4°C. Pellets were briefly rinsed with 1 mL of TEVP buffer and resuspended in 1 mL TEVP buffer. These synaptosomal fractions were sonicated for 10 seconds and the protein concentration was measured by Bradford assay with bovine serum albumin as standards. Synaptosomal fractions were diluted with water containing Complete Mini Protease Inhibitor Cocktail (Roche) and mixed with equal volume of 2× SDS-PAGE loading buffer to the final protein concentration of 0.5 µg/µL. The solution was then boiled for 5 min, incubated on ice for 1 min, and centrifuged for 5 min to obtain the supernatant for loading at 10 µg each lane. In both fractions, the separated proteins were transferred to the nitrocellulose membrane. The membrane was blocked with 5% milk in wash buffer and treated with the primary antibody of the protein of interest. The secondary antibodies and detecting reagents were the same as previously described. The experiments were performed in triplicate. For whole hippocampus fraction the antibody used was against β-galactosidase (55976, MP Biomedicals) and for synaptosomal fraction the antibodies used were against syntaxin-1 (PA1-1042, Affinity BioReagents), SNAP-25 (SC-7538, Santa Cruz), synaptotagmin-1 (AB9202, Millipore), synaptobrevin-2 (018-15791, Wako), synaptophysin (PA1-1043, Affinity BioReagents), and dynamin 1 (SC-6402, Santa Cruz).

### Statistics

Long-term potentiation (LTP) and paired-pulse ratios (PPRs) were analyzed using a Student's t-test. Input-output relationships were analyzed at individual intensities using a Student's t-test and the overall distribution was analyzed using a Kolmogorov-Smirnov test. Miniature excitatory post-synaptic current (mEPSC) amplitude and frequency was analyzed using a Kolmogorov-Smirnov test, while rise and decay times were analyzed using a Student's t-test. Graphical data shows approximately 97–98% of mEPSC data for simplicity. Western blot analyses were conducted using a Student's t-test. Fear conditioning behavior response to the shock during training was analyzed using Wilcoxon rank-sum test, using SAS 9.1 software. Shock threshold was analyzed for each of three behaviors using a Student's t-test. Fear conditioning freezing analysis was conducted by mixed-model ANOVA taking into consideration age, sex, and bodyweight interactions, using SAS 9.1 software. Significance was assigned at p≤0.05.

## Results

**Figure 2:**
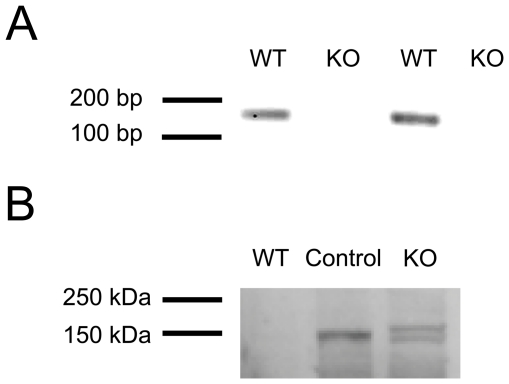
Confirmation of loss of WT mRNA and fusion protein expression in mutant hippocampus. (**A**) The WT mice, in contrast to the homozygous *Btbd9* mutant mice, produced a strong RT-PCR fragment of the Btbd9 gene. (**B**) The Btbd9 protein is expressed in the hippocampus of adult mice. Antibody against β-galactosidase detected β-geo fusion proteins from both the *Btbd9* mutant and a positive control mouse that had a gene trap insertion in a different gene, while no band was observed in the WT control.

To examine the function of the Btbd9 protein *in vivo*, we generated a knockout of the *Btbd9* gene in mice using a β-geo gene trap vector inserted into the sixth intron. Interbreeding heterozygous *Btbd9* mutant mice resulted in a non-Mendelian ratio at weaning, with a decrease in both heterozygous and homozygous *Btbd9* mutant mice (p<0.05, Table 1). There were no obvious abnormalities in these mice, including weight at time of experiment, ability to right themselves, and gait. To determine how effective the gene trap was in terminating transcription, we used reverse transcriptase (RT)-PCR with the forward primer located in the exon before the gene trap and the reverse primer located in the exon after the gene trap. The wild-type (WT) mice, in contrast to the homozygous *Btbd9* mutant mice, produced a strong RT-PCR fragment (Figure 2A). This suggests that the gene trap efficiently knocked out the *Btbd9* gene. Furthermore, as there are currently no high-quality commercially available antibodies for Btbd9 in mice, we took advantage of the mutant mice having the bacterial *lacZ* gene inserted into the *Btbd9* gene as part of the gene trap, which produces a β-galactosidase-neomycin fusion protein. Using an antibody against β-galactosidase, we were able to identify that Btbd9 is normally expressed in the hippocampus of mice (Figure 2B). The *Btbd9* mutant mice produced two bands compared to one band by the positive control, which could be attributed to alternate splicing of the *Btbd9* gene [29]. The expression of the *Btbd9* gene in the hippocampus of mice is similar to the result produced by the Allen Mouse Brain Atlas using *in situ* hybridization of *Btbd9* mRNA [13].

**Table 1:** Non-Mendelian ratios of pups from heterozygous Btbd9 mutant mice interbreeding.

Genotype	Expected	Observed	Chi-square value	p-value
WT	20.5	35	-	-
Het	41	34	9.391	0.0022*
KO	20.5	13	10.083	0.0015*
Total	82	82	14.195	0.0008**

WT, Wild-type mice; Het heterozygous *Btbd9* mutant mice; KO, homozygous *Btbd9* mutant mice;

* p<0.05,

** p<0.001.

**Figure 3:**
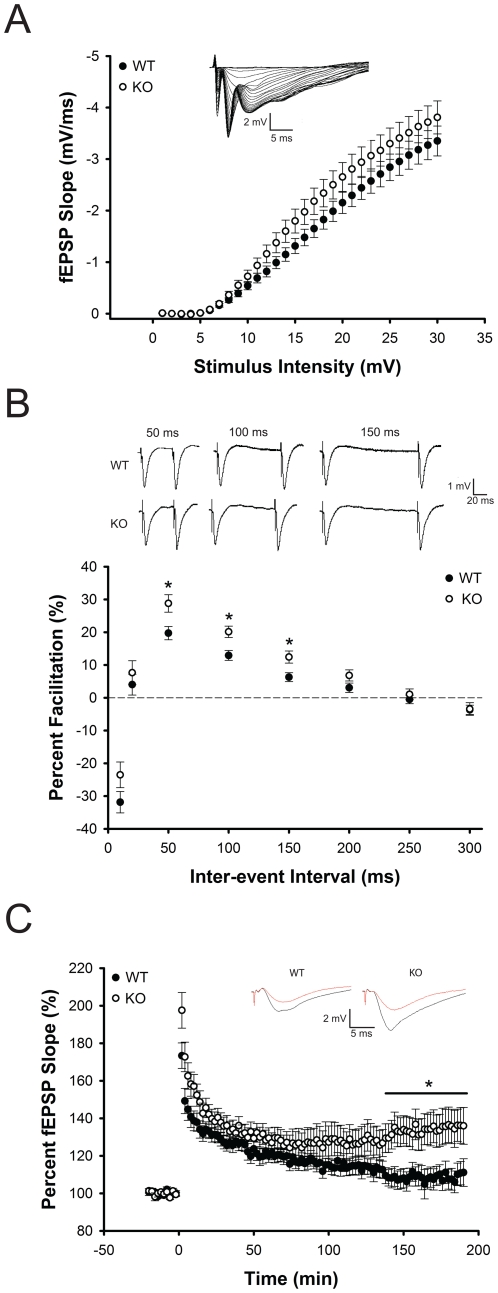
Hippocampal CA1 electrophysiological field recordings. (**A**) *Btbd9* mutant mice showed no differences in input-output relationships. Small inset graph is a representative trace with varying stimulus intensities. (**B**) *Btbd9* mutant mice showed enhanced paired-pulse ratios at three inter-stimuli values. Panel above graph are representative traces at the three inter-stimuli values that were significantly different between *Btbd9* mutant and WT mice. (**C**) *Btbd9* mutant mice showed enhanced late long-term potentiation. Small inset graphs are representative traces, with red signifying before LTP induction and black signifying after LTP induction. Circles represent means ± SEM. * p<0.05.

**Figure 4:**
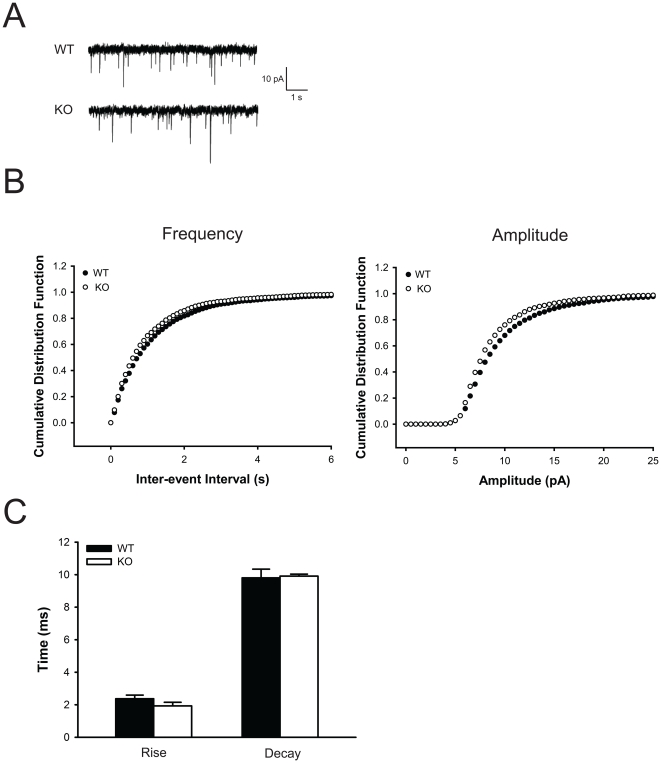
Hippocampal CA1 electrophysiological whole-cell recordings. (**A**) Representative traces of mEPSC recording from WT and *Btbd9* mutant mice. (**B**) *Btbd9* mutant mice showed a decrease in both frequency and amplitude of mEPSC events compared to WT mice (p<0.01 each). (**C**) *Btbd9* mutant mice showed no difference from WT in rise and decay times of mEPSC events. Bars represent means ± SEM.

As the hippocampus has been implicated in learning and memory and extensively studied using electrophysiological techniques, we performed field recordings in hippocampal slices in the CA1 region to determine whether the *Btbd9* mutant mice have any electrophysiological alterations. To test for post-synaptic deficits in the *Btbd9* mutant mice, we obtained input-output curves, which measure the post-synaptic potential slope versus varying stimulus intensities. *Btbd9* mutant mice showed no change in their input-output relationship at individual stimulus intensity values and no change in the distribution of the curve compared to WT mice (p>0.05; Figure 3A). Next, to examine short-term plasticity in the *Btbd9* mutant mice we looked at paired-pulse ratios (PPRs), which test the ability of two stimuli separated by varying time intervals to elicit an increased post-synaptic response. PPRs at three different inter-stimulus intervals were significantly enhanced in the *Btbd9* mutant mice compared to WT mice (p<0.05; Figure 3B). This suggests that the *Btbd9* mutant mice had a decrease in the probability of synaptic vesicle release from the pre-synaptic terminal. Next, to examine long-term plasticity in the *Btbd9* mutant mice we examined long-term potentiation (LTP). *Btbd9* mutant mice compared to WT mice had a significant enhancement in late CA1 LTP (p<0.05; Figure 3C). This suggests that the *Btbd9* mutant mice had an enhancement in long-term plasticity. Lastly, to further explore these alterations, we examined miniature excitatory post-synaptic currents (mEPSC) for alterations in amplitude, frequency, and rise and decay times. We found that the *Btbd9* mutant mice have decreased frequency and amplitude of mEPSC events compared to WT mice (p<0.01, Figure 4B), but no alteration in the rise and decay times (p>0.05, Figure 4C). Decreased frequency suggests that the number of vesicles released at rest is decreased, while decreased amplitude suggests that the amount of glutamate per synaptic vesicle, the post-synaptic response to a single synaptic vesicle, or both, could be decreased. Additionally, the data suggest that there is no change in the kinetics of the post-synaptic receptors, such as opening and closing of their respective channels.

**Figure 5:**
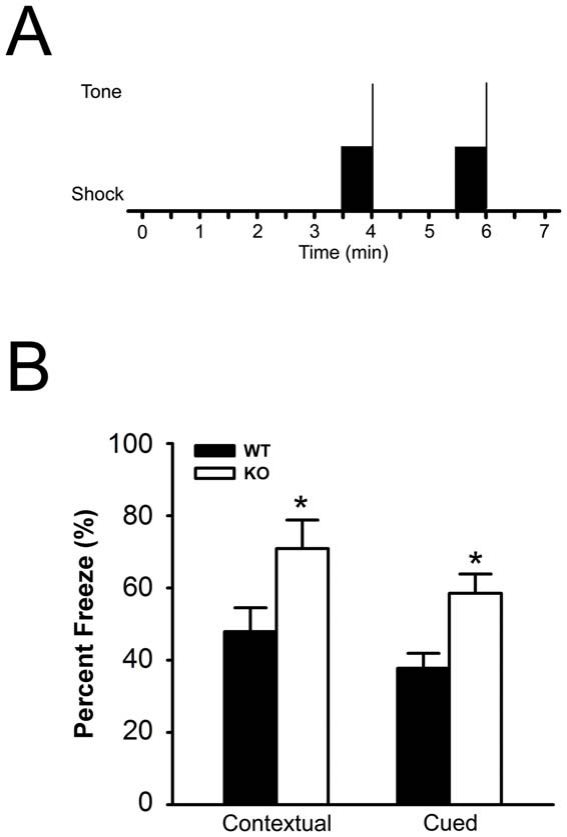
Freezing behavior in fear conditioning experiment. (**A**) *Btbd9* mutant mice and WT mice were conditioned to two 30-second tones followed by an electric shock, with a shock interval of 120 seconds. Solid black rectangle represents the period of tone. The vertical line at represents the period of the shock. (**B**) *Btbd9* mutant mice had increased percentage of freezing behavior in both contextual and cued fear conditioning, suggesting that the *Btbd9* mutant mice had enhanced fear memory. Vertical bars represent means ± SEM. * p<0.05.

As alterations in hippocampal long-term potentiation have been associated with alterations in hippocampal learning and memory, we performed a classical fear conditioning to test both cued fear memory, freezing behavior caused by an auditory stimulus, and contextual fear memory, freezing behavior caused by recall of the environment or context (Figure 5A). The *Btbd9* mutant mice exhibited no behavioral response difference to the shock during the training period (p>0.05, Figure S2B). Moreover, the *Btbd9* mutant mice had no difference in the electrical shock required to elicit flinching, jumping, and vocalization behaviors (p>0.05, Figure S2A). This suggests that the *Btbd9* mutant mice do not have altered perception to the electrical shock. However, we found that the *Btbd9* mutant mice had an enhancement of both cued (p<0.05, Figure 5B) and contextual (p<0.05, Figure 5B) fear memory. This suggests that the *Btbd9* mutant mice have an enhancement in learning and memory that correlates with the enhanced LTP.

**Figure 6:**
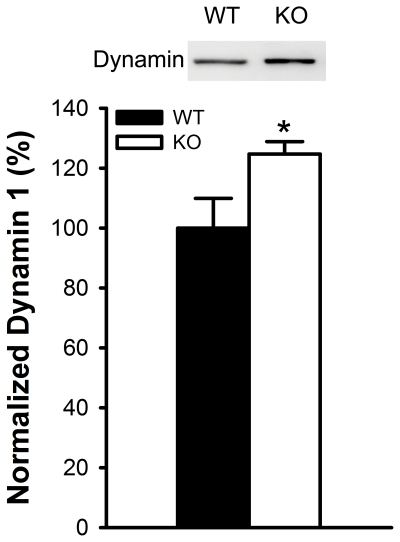
Dynamin 1 levels in the synaptosomal fraction of the hippocampi of *Btbd9* mutant mice. Image is a representative Western blot analysis of dynamin in WT and *Btbd9* mutant mice. Bar graph is quantitative analysis of the Western blot, showing a significant increase in dynamin 1 in the *Btbd9* mutant mice. Bars represent means ± SEM. * p = 0.05.

To explore the molecular basis for the pre-synaptic alteration in the *Btbd9* mutant mice, we performed Western blot analyses to determine the level of several proteins involved in the Soluble NSF Attachment Protein Receptor (SNARE) complex, which are involved in synaptic vesicle docking, fusion, and endocytosis. We first determined the levels of the target-SNAREs syntaxin-1 and SNAP-25 and the vesicular-SNAREs synaptotagmin-1 and synaptobrevin-2. We also examined the levels of endocytosis proteins synaptophysin and dynamin 1. We found no statistical difference in the protein levels of synataxin-1, SNAP-25, synaptobrevin-2, synaptotagmin-1, or synaptophysin suggesting there is no functional alteration in vesicular docking or fusion events (p>0.05, Figure S3A-E). However, there was a significant increase in the level of dynamin 1 in the *Btbd9* mutant mice (p<0.05, Figure 6).

## Discussion

To understand the function of the BTBD9 protein *in vivo* and its potential role in the pathophysiology of restless legs syndrome (RLS), we generated a knockout of the *Btbd9* gene, the mouse homolog of the human *BTBD9* gene, and found that this protein is expressed in the hippocampus. We further found that the homozygous *Btbd9* mutant mice exhibited alterations in hippocampal paired-pulse ratios (PPRs), a measure of short-term plasticity, and hippocampal long-term potentiation (LTP), which is a measure of long-term plasticity. Additionally, we found that the homozygous *Btbd9* mutant mice exhibited decreased frequency and amplitude of miniature EPSC (mEPSC) events. Furthermore, the homozygous *Btbd9* mutant mice had enhanced cued and contextual fear memory. Lastly, the homozygous *Btbd9* mutant mice had an increase in the levels of dynamin 1, an enzyme associated with endocytosis.

We first examined the electrophysiological characteristics of the Schaffer collateral CA3-CA1 pyramidal neurons, which use glutamate as its neurotransmitter, in the *Btbd9* mutant mice. We found that the *Btbd9* mutant mice have an enhanced late long-term potentiation. LTP is a measure of long-term synaptic transmission and strength between pre- and post-synaptic neurons on the order of hours to days and is believed to underlie the learning and memory process [30], [31]. LTP can be broken into two temporal categories – early and late. It is hypothesized that early LTP is modulated by properties of the pre- and post-synaptic neurons, such as resting membrane potentials, voltage-gated calcium channels, and neurotransmitter receptors. Late LTP, unlike early LTP, is believed to be protein synthesis-dependent due to its extended time period [32], [33]. This is done through alterations in gene expression, possibly through transcription factors and kinases such as cAMP response element binding protein (CREB) and the mitogen-activated protein (MAP) kinase family [34]. To examine the effects of this alteration in late LTP in the *Btbd9* mutant mice, we measured cued and contextual fear memory. While fear memory is generally thought to be controlled by the amygdala, there is an extensive body of information supporting the role of the hippocampus in fear memory, in particular contextual fear memory [35]–[39]. We have found that the *Btbd9* mutant mice have an enhanced cued and contextual fear memory. In restless legs syndrome, patients often have abnormal sensations in their legs during rest and at night. Furthermore, a recent report showed that there is a decrease in pain threshold and temporal summation of heat pain in RLS patients [40]. The authors suggest that this could be attributed to an alteration in the central nervous system pain processing centers, which could lead to persistent pain conditions [40]. Recent studies have also suggested that an enhanced synaptic plasticity in nociceptive pathways can cause heightened pain awareness [41]–[44]. In particular, nociceptive nerves can undergo long-term potentiation, which has been suggested as a possible mechanism underlying hyperalgesia [45]. Therefore, we speculate that enhanced synaptic plasticity or potentiation, as seen here in the hippocampus, could be present elsewhere in the *Btbd9* mutant mice, such as sensory or nociceptive centers in the central nervous system, and underlie potential sensory and nociceptive deficits in RLS patients. Further studies will need to be conducted to examine this.

Furthermore, in the electrophysiological recordings we found that the *Btbd9* mutant mice had enhanced paired-pulse ratios, a measure of neural facilitation, which is the ability of a second impulse to evoke the further release of synaptic vesicles from the pre-synaptic terminal. PPRs are on the order of milliseconds and seconds and therefore a measure of short-term plasticity. PPRs have been associated with an inverse probability of synaptic vesicle release, where increases in PPRs are indicative of a decreased probability of synaptic vesicle release and vice versa. We therefore tested proteins that are involved in synaptic transmission and plasticity. We found that the *Btbd9* mutant mice have an elevated level of dynamin 1. Dynamin is a GTPase associated with the cleavage of vesicles from the membrane during endocytosis [46]. A previous study showed that dynamin 1 knockout mice have a decrease in synaptic vesicle recycling, possibly due to an inability of vesicles to free themselves from the membrane [47]. Additionally, in a recent study decreases in dynamin levels were associated with memory impairment in a rat model of Alzheimer's disease [48]. Furthermore, treatment of these rats with memantine, a classic NMDA antagonist used to treat Alzheimer's disease, resulted in decreased dynamin 1 degradation and increased memory ability [48]. This study closely correlates with our results by showing a relation between dynamin and learning and memory.

A recent proteomic study examining the ubiquitination system identified 774 candidate interacting proteins associated with deubiquitinating enzymes [49]. Of these, BTBD9 was associated with ubiquitin specific peptidase 21 (USP21) and COP9 constitutive photomorphogenic homolog subunit 6 (CSN6) [49]. In addition, the BTBD9 protein is known to have a BACK domain, which is important in substrate orientation for ubiquitin complexes [5]. These data suggests that BTBD9 might be involved in the ubiquitination system. We speculate that BTBD9 through its interactions with USP21 and CSN6 plays a role in the turnover of dynamin 1. Therefore, in the *Btbd9* mutant mice, the loss of the Btbd9 protein could lead to a decreased degradation of dynamin 1, which results in an elevated level of dynamin 1, as observed. Dynamin 1 thereby can alter the characteristics of synaptic function, such as long-term potentiation, which can influence memory performance.

The *Btbd9* mutant mice have enhanced PPRs, which is suggestive of a pre-synaptic impairment, but have no alteration in input-output relationships, in which a pre-synaptic neuron is stimulated at varying intensities and the response of a post-synaptic neuron is recorded. We speculate that either in the *Btbd9* mutant mice there is a post-synaptic enhancement, which compensates for a pre-synaptic impairment; or, the input-output relationship measurement is not sensitive enough to measure the effects of the pre-synaptic deficit on the post-synaptic neuron. To potentially differentiate between these two, we measured mEPSC events. In this experiment action potentials from the pre-synaptic neurons are blocked, and only spontaneous, action potential independent vesicular release is allowed. We observed that the *Btbd9* mutant mice have decreased frequency and amplitude of mEPSC events. Decreased frequency suggest that there is a decrease in synaptic vesicle release at rest, which correlates with the PPRs, while decreased amplitude suggest a decrease in quantal size of vesicular glutamate, a decrease in unit post-synaptic response, or both. It is worth noting that it has been suggested that evoked responses, such as input-output relationships, and spontaneous responses, such as mEPSC, may not be directly compared due to different synaptic mechanisms underlying them [50]. Further examination of the pre-synaptic and post-synaptic terminals will be conducted in future studies.

Furthermore, in the *Btbd9* mutant mice there is an enhancement of late long-term potentiation, while there is no change in early long-term potentiation. We speculate that there is no change in early long-term potentiation either because the *Btbd9* mutant mice have an alteration in the post-synaptic neuron that can compensate for pre-synaptic deficits, or *Btbd9* as a protein involved in the ubiquitination system is altering the longevity of proteins involved in synaptic plasticity. Additionally, late long-term potentiation requires changes in protein synthesis, which we speculate carries different properties than that of the PPRs. Further examination of the post-synaptic terminal and other proteins involved in synaptic plasticity will need to be conducted to test these hypotheses.

In conclusion, the function of the BTBD9 protein is poorly understood and no previous study has been designed to specifically understand its normal function and potential role in the pathophysiology of RLS. Here, we attempt to tackle this issue by generating a line of *Btbd9* mutant mice. Furthermore, using this line of mutant mice, we have found that this protein regulates hippocampal synaptic plasticity and fear memory. Further research will need to be conducted to examine whether other brain regions show similar abnormalities and the potential role of BTBD9 in RLS.

## Supporting Information

Figure S1 **Location of the β-geo vector insertion site and PCR map.** (**A**) Schematic diagram of PCR reactions that were used to narrow the β-geo vector insertion site to between 102,250 and 102,501. Reaction 1 produced PCR products at the appropriate base pair (bp) length, whereas Reaction 2 failed to produce reactions of the appropriate size, thereby narrowing the location to between 102,000 to 102,501. This was further confirmed by Reaction 3, which failed to produce reactions of the appropriate size. This region was further broken down, with Reactions 4 and 5. Reaction 4 produced PCR products at the appropriate bp length, whereas Reaction 5 failed to produce reactions of the appropriate size, thereby narrowing the location to 102,200 to 102,501. (**B**) Schematic diagram of PCR primer locations used to genotype mice. A set of primers detects the wild-type allele, and a different set of primers detects the mutant allele. Base pair numbers are relative to the start of the sixth intron of the *Btbd9* gene. Thick line represents β-geo gene trap, and thin line represents the sixth intron of the *Btbd9* gene.
(TIF)

Figure S2 **Response to electric shocks.** (**A**) *Btbd9* mutant mice showed no difference in their threshold to electrical shock in any of three measured behaviors – flinching, jumping, or vocalization. (**B**) *Btbd9* mutant mice showed no difference in behavioral response on a rating scale of 1 to 5 to either of the electric shocks during the training phase of the fear conditioning experiment. (**C**) *Btbd9* mutant mice showed no difference in freezing behavior in the first 3 minutes of the cued fear conditioning test. Bars represent means ± SEM.
(TIF)

Figure S3 **Western blot analyses of synaptic proteins from hippocampal synaptosome fractions.** Representative Western blot images and quantitative analysis of SNAP-25 (**A**), syntaxin (**B**), synaptotagmin (**C**), synaptophysin (**D**), and synaptobrevin (**E**). Bars represent means ± SEM.
(TIF)

Table S1 **Primers located in the sixth intron of the** ***Btbd9*** **gene.** Length is the number of nucleotides. Locations are relative to the start of Intron 6 (intron between Exons 6 and 7).
(XLSX)

Table S2 **Primers located in the β-geo gene trap vector.** Length is the number of nucleotides.
(XLSX)
