# Supplementary figures and images for: Enhanced Hippocampal Long-Term Potentiation and Fear Memory in Btbd9 Mutant Mice
Source: PLoS One. 2012 Apr 19;7(4):e35518. doi: 10.1371/journal.pone.0035518 (PMC3334925; doi:10.1371/journal.pone.0035518)

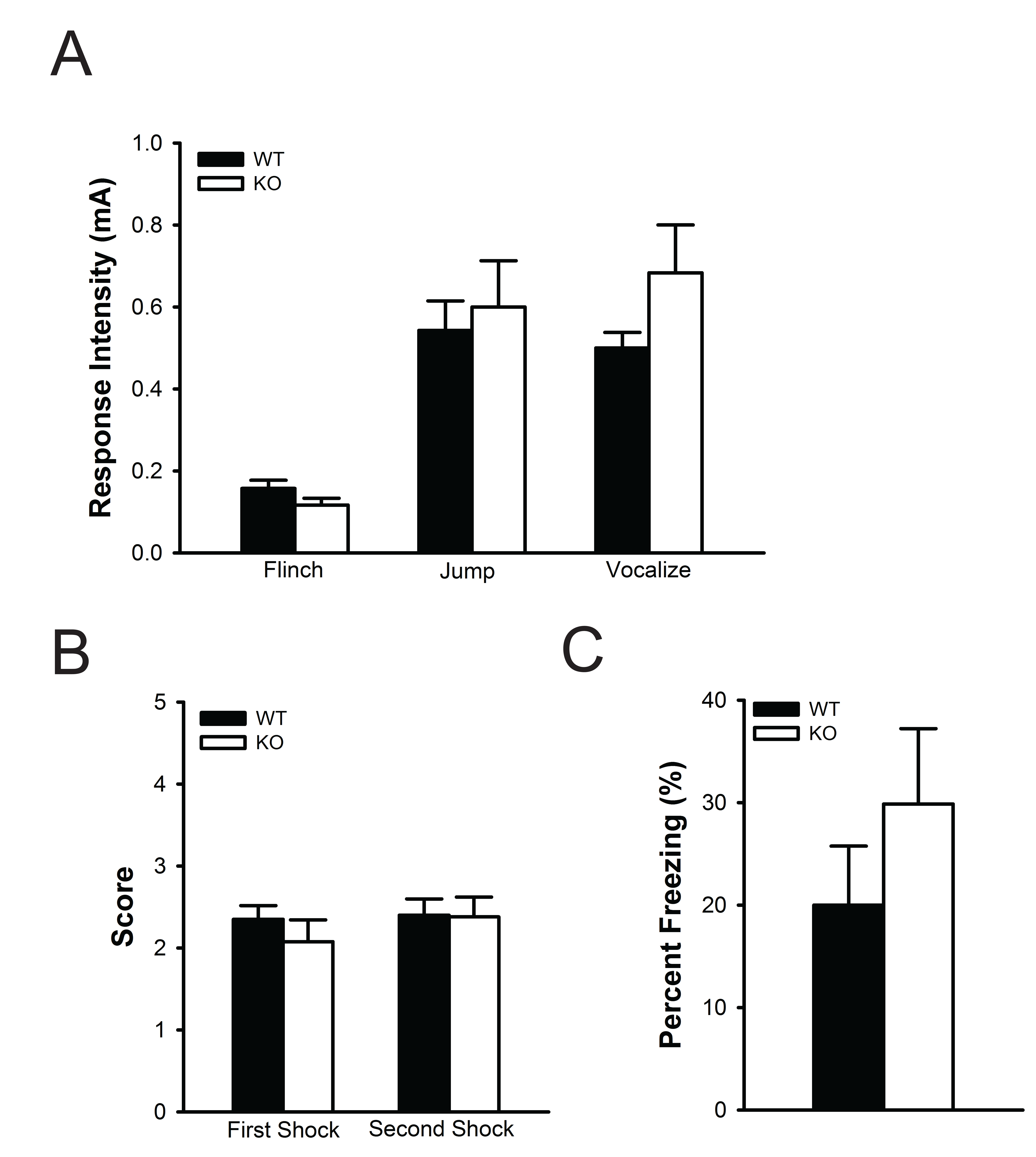

Supplement: Figure S2 — Response to electric shocks. (A) Btbd9 mutant mice showed no difference in their threshold to electrical shock in any of three measured behaviors – flinching, jumping, or vocalization. (B) Btbd9 mutant mice showed no difference in behavioral response on a rating scale of 1 to 5 to either of the electric shocks during the training phase of the fear conditioning experiment. (C) Btbd9 mutant mice showed no difference in freezing behavior in the first 3 minutes of the cued fear conditioning test. Bars represent means ± SEM. (TIF) [file pone.0035518.s002.tif]

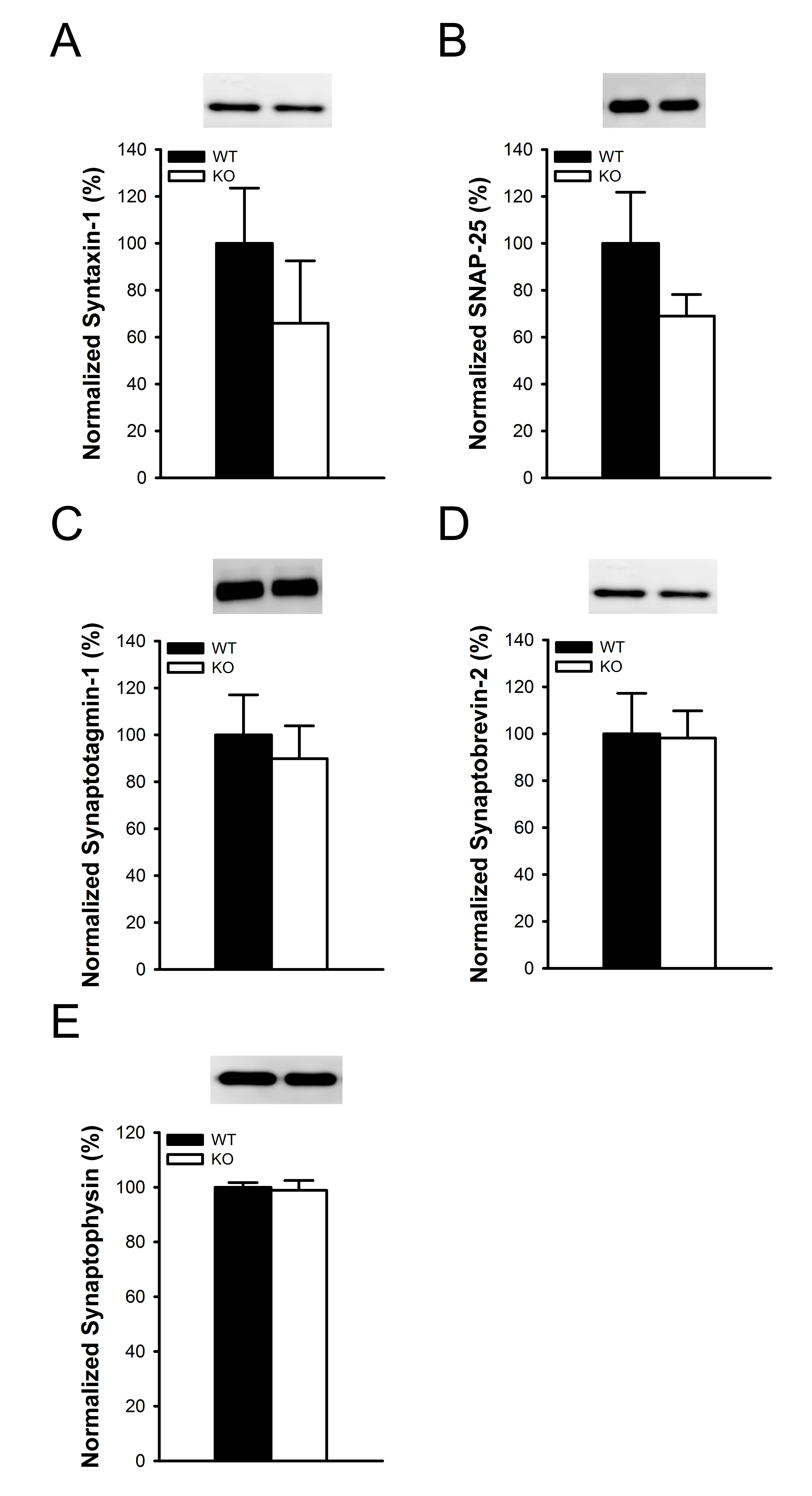

Supplement: Figure S3 — Western blot analyses of synaptic proteins from hippocampal synaptosome fractions. Representative Western blot images and quantitative analysis of SNAP-25 (A), syntaxin (B), synaptotagmin (C), synaptophysin (D), and synaptobrevin (E). Bars represent means ± SEM. (TIF) [file pone.0035518.s003.tif]
